# Complete chloroplast genomes of four *Physalis* species (Solanaceae): lights into genome structure, comparative analysis, and phylogenetic relationships

**DOI:** 10.1186/s12870-020-02429-w

**Published:** 2020-05-28

**Authors:** Shangguo Feng, Kaixin Zheng, Kaili Jiao, Yuchen Cai, Chuanlan Chen, Yanyan Mao, Lingyan Wang, Xiaori Zhan, Qicai Ying, Huizhong Wang

**Affiliations:** 1 College of Life and Environmental Science, Hangzhou Normal University, Hangzhou, 311121 China; 2 Zhejiang Provincial Key Laboratory for Genetic Improvement and Quality Control of Medicinal Plants, Hangzhou Normal University, Hangzhou, 311121 China; 3 College of Bioscience & Biotechnology, Hunan Agricultural University, Changsha, 410128 China

**Keywords:** *Physalis*, Chloroplast genome, Molecular markers, Species identification, Phylogenetic relationship

## Abstract

**Background:**

*Physalis* L. is a genus of herbaceous plants of the family Solanaceae, which has important medicinal, edible, and ornamental values. The morphological characteristics of *Physalis* species are similar, and it is difficult to rapidly and accurately distinguish them based only on morphological characteristics. At present, the species classification and phylogeny of *Physalis* are still controversial. In this study, the complete chloroplast (cp) genomes of four *Physalis* species (*Physalis angulata*, *P. alkekengi* var. *franchetii*, *P. minima* and *P. pubescens*) were sequenced, and the first comprehensive cp genome analysis of *Physalis* was performed, which included the previously published cp genome sequence of *Physalis peruviana*.

**Results:**

The *Physalis* cp genomes exhibited typical quadripartite and circular structures, and were relatively conserved in their structure and gene synteny. However, the *Physalis* cp genomes showed obvious variations at four regional boundaries, especially those of the inverted repeat and the large single-copy regions. The cp genomes’ lengths ranged from 156,578 bp to 157,007 bp. A total of 114 different genes, 80 protein-coding genes, 30 tRNA genes, and 4 rRNA genes, were observed in four new sequenced *Physalis* cp genomes. Differences in repeat sequences and simple sequence repeats were detected among the *Physalis* cp genomes. Phylogenetic relationships among 36 species of 11 genera of Solanaceae based on their cp genomes placed *Physalis* in the middle and upper part of the phylogenetic tree, with a monophyletic evolution having a 100% bootstrap value.

**Conclusion:**

Our results enrich the data on the cp genomes of the genus *Physalis*. The availability of these cp genomes will provide abundant information for further species identification, increase the taxonomic and phylogenetic resolution of *Physalis*, and assist in the investigation and utilization of *Physalis* plants.

## Background

The genus *Physalis* L., consisting of 75–120 species, is a well-known genera of the family Solanaceae because of its significant economic value, owing to the medicinal, edible and ornamental uses of its members [1–3]. It is mainly distributed in the tropical and temperate Americas, with only few species are found in Eurasia and Southeast Asia [1, 4–6]. China has approximately five species and two varieties of *Physalis* plants, which were used as medicinal herbs for more than 2000 years by the Chinese people. Many *Physalis* species have a variety of pharmacological activities, leading to anti-inflammatory, anti-oxidant, and anti-cancer benefits, and are used to treat many illnesses, including malaria, rheumatism, hepatitis, asthma, cancer, and liver disorders [2, 7–11]. The Pharmacopoeia of the People’s Republic of China included *Physalis alkekengi* var. *franchetii* as a standard *Physalis* medical plant in 2015 [7]. Moreover, many *Physalis* species, such as *P. pubescens*, *P. peruviana*, *P. alkekengi* var. *franchetii*, and *P. philadelphica*, are cultivated in many regions of the world for their edible fruit or as ornamental plants [4, 12].

The chloroplast (cp) is an important organelle in plant cells and plays an important role in many plant cell functions, such as photosynthesis, carbon fixation, and stress response [13, 14]. In most plants, the cp genome’s structure is very conservative, being circular with a length of 120–170 kb, including four typical areas: two inverted repeats (IRs), large single-copy (LSC) and small single-copy (SSC) regions [15]. In a cp genome, the gene content and gene composition are highly conserved, generally containing 120–130 genes [16]. In addition, the evolutionary rate of a cp genome is usually slow compared with nuclear DNA sequence [17]. However, some significant structural genomic changes, including gene losses, large inversions, and contraction or expansion of IR regions, have been observed during the evolution of the cp genomes of some angiosperms [16–18]. For example, the *infA*, *rpl22*, *rpl33*, *rps16*, *ycf1*, *ycf2*, *ycf4* and *accD* genes have been lost in some plant species [16, 19–21]. Furthermore, the IR regions of some species, such as *Pisum sativum* [22], *Glycine max* [23], *Crytomeria japonica* [24], *Taxus chinensis* var. *mairei* [25], and *Vigna radiata* [26] showed complete or partial losses. These cp genomic differences may be the results of differential indels and substitutions rates during the evolution of plant species [27]. Owing to the conserved structure, moderate evolutionary rates, and uniparental inheritance of cp genomes, the sequences are often used as genetic markers for DNA barcoding, and phylogenetic and evolutionary studies [17, 28–30].

In recent years, because of their various significant commercial values, the taxonomy of *Physalis* has become a concern, and its characterization is regarded as one of the most challengingly in Solanaceae [1, 3, 31, 32]. Traditionally, the genus *Physalis* was divided into species groups by morphological and/or geographical characters, such as habit, hair type, and number of calyx angles [5, 31]. Lately, with the raise of molecular taxonomy, the ribosomal internal transcribed spacer (ITS) 1 and ITS2, chloroplast *ndhF*, *trnL-F* and *psbA-trnH* sequences, and *Waxy* genes, have been used in species identification and phylogenetic analyses of *Physalis*, as well as to determine their relationship to other genera in the Solanaceae family [1, 3, 32, 33]. In addition, some DNA marker systems, including simple sequence repeat (SSR), inter-simple sequence repeats, and sequence-characterized amplified region markers, have been used in the genetic study of *Physalis* plants [4, 12, 34, 35]. However, owing to the limited information on these traditional genetic markers, there are still some controversies regarding the species identification and taxonomy of *Physalis* [3, 28]. The application and development of the cp genome in plant phylogenetic studies provide a new research idea for the better study of phylogenetic classification of *Physalis*. Advances in next-generation sequencing techniques have facilitated rapid progress in the field of cp genomics [36, 37]. By September 2019, more than 3000 complete cp genome sequences, including *P. peruviana* (GenBank accession number: NC_026570) as sole representative of *Physalis* genus without further analysis or study, were released into the National Center for Biotechnology Information (NCBI) organelle genome database (https://www.ncbi.nlm.nih.gov/genome/organelle/).

Here, we sequenced the cp genomes of four *Physalis* species (*P. angulata*, *P. alkekengi* var. *franchetii*, *P. minima* and *P. pubescens*), and performed an in deep analysis of the genomes, representing the first comprehensive analysis of cp genomes of *Physalis*, including the previously released *P. peruviana* cp genome. Our study’s aims were: (1) to present the complete cp genome sequences of four *Physalis* species; (2) to characterize and compare the global structural patterns of available *Physalis* cp genomes; (3) to examine variations in the SSRs and repeat sequences among the five *Physalis* cp genomes; and (4) to improve our understanding of the evolutionary and systematics positions of the genus *Physalis* within Solanaceae based on their cp genome sequences.

## Results

### Overall genome sequencing and assembly

Total genomic DNA was extracted from ~ 0.1 g of a six individuals pool of healthy, clean and fresh leaves per each *Physalis* species (Additional File 1: Table S1), and used to generate the corresponding Illumina MiSeq libraries by long-range PCR (see Methods section). After Illumina sequencing (paired-end, 250x), reads were QC filtered, mapped against *P. peruviana* cp reference genome (NC_026570) and assembled to obtain the four complete cp genomes. Clean bases mapped to the *P. peruviana* cp reference genome, with mean coverages ranging from 480x to 1756x (Additional File 1: Table S2).

### *Physalis* cp genome features

**Fig. 1:**
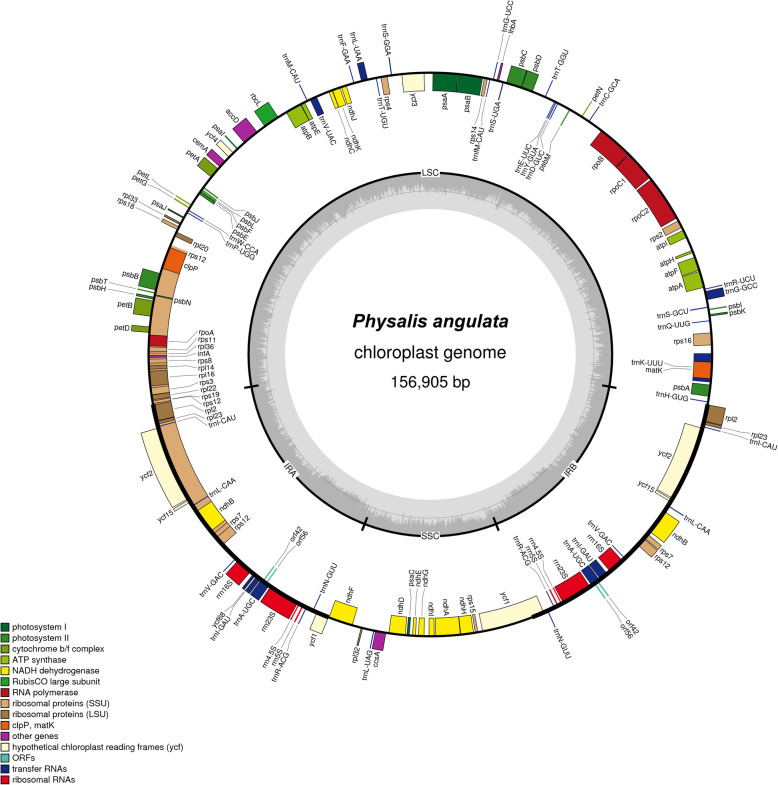
Gene map of the *P. angulata* chloroplast genome. Genes shown outside the outer circle are transcribed clockwise, and those inside are transcribed counterclockwise. Genes belonging to different functional groups are color coded. The darker gray in the inner circle indicates the GC content, and the lighter gray indicates the AT content. The inner circle also indicates that the chloroplast genome contains two copies of the inverted repeat (IRA and IRB), a large single-copy region (LSC) and a small single-copy region (SSC). The map was constructed using OrganellarGenomeDRAW

The full length of *Physalis* cp genomes ranged from 156,578 bp (*P. alkekengi* var. *franchetii*) to 157,007 bp (*P. pubescens*) (Table 1). The gene maps of the newly sequenced *Physalis* cp genomes were provided in Fig. 1 (*P. angulata*) and in Additional File 2: Fig. S1–S3 (*P. alkekengi* var. *franchetii*, *P. minima* and *P. pubescens*). Like most angiosperms, the *Physalis* cp genomes also exhibited the typical quadripartite structure, distributed in one LSC region (86,845 bp–88,309 bp), one SSC region (18,363 bp–18,503 bp), and a pair of IR regions (A and B; 24,953 bp–25,685 bp). The overall GC content of each cp genome was comparable, ranging from 37.52 to 37.65%. Whereas the GC content was distributed differentially between each region, showing greater GC content at IR regions than in the LSC or SSC (Table 1). Compared with *P. peruviana*, the new cp genomes contained 2 more genes each (total genes 132 vs 130), some of them found in duplicate generally located at the IR regions (see Table 1). When duplicated genes in the IR regions were counted only once, each of the new four cp genomes (*P. angulata*, *P. alkekengi var. franchetii*, *P. minima*, and *P. pubescens*) contained the same 114 genes, distributed as 80 protein-coding genes, 4 rRNA genes, and 30 tRNA genes. While the *P. peruviana* cp genome contained only 113 genes, missing a protein-coding gene. These 114/113 genes encode for self-replication-related functions, photosynthesis-related, and other proteins, and as well as unknown proteins (Table 2). Of these 114/113 genes, 17 are intron-containing genes, 15 that contain one intron (*rpl2*, *rpl16*, *rpoC1*, *rps12*, *rps16*, *trnA-UGC*, *trnG-GCC*, *trnI-GAU*, *trnK-UUU*, *trnL-UAA*, *trnV-UAC*, *atpF*, *ndhA*, *ndhB*, and *petB*) and two that contain two introns (*clpP* and *ycf3*).

**Table 1:** Summaries of complete chloroplast genomes of five *Physalis* species

	*P. angulata*	*P. alkekengi* var. *franchetii*	*P. minima*	*P. pubescens*	*P. peruviana*
Genome size (bp)	156,905	156,578	156,692	157,007	156,706
Large single copy (LSC, bp)	87,108	88,309	86,845	87,137	86,995
Small single copy (SSC, bp)	18,469	18,363	18,503	18,500	18,393
Inverted repeat (IR, bp)	25,664	24,953	25,672	25,685	25,659
GC content (%)					
Total genome	37.52	37.65	37.54	37.53	37.54
LSC	35.58	35.76	35.60	35.59	35.57
SSC	31.32	31.72	31.40	31.35	31.36
IR	43.05	43.20	43.03	43.06	43.08
Gene (total /different)	132/114	132/114	132/114	132/114	130/113
genes duplicated in IR	18	18	18	18	17
protein-coding genes (total/in IR)	87/7	87/7	87/7	87/7	85/6
rRNA (total/different)	8/4	8/4	8/4	8/4	8/4
tRNA (total/different)	37/30	37/30	37/30	37/30	37/30
GenBank accession	MH045574	MH045575	MH045577	MH045576	NC_026570
References	This study	This study	This study	This study	Genbank

**Table 2:** Genes in the *Physalis* chloroplast genomes

Category for genes	Group of genes	Name of genes
Self-replication	Large subunit of ribosome	①*rpl2(×2), *rpl14*, *rpl16, *rpl20*, *rpl22*, *rpl23*(× 2), *rpl32*, *rpl33*, *rpl36*
	DNA dependent RNA polymerase	*rpoA*, *rpoB*, *rpoC1, *rpoC2*
	Small subunit of ribosome	*rps2*, *rps3*, *rps4*, *rps7*(×2), *rps8*, *rps11*, *rps12(× 2), *rps14*, *rps15*, *rps16, *rps18*, *rps19*
	rRNA Genes	*rrn4.5S*(×2), *rrn5S*(× 2), *rrn16S*(× 2), *rrn23S*(× 2)
	tRNA Genes	*trnA-UGC(×2), *trnC-GCA*, *trnD-GUC*, *trnE-UUC*, *trnF-GAA*, *trnfM-CAU*, *trnG-GCC, *trnG-UCC*, *trnH-GUG*, *trnI-GAU(× 2), *trnI-CAU*(× 2), *trnK-UUU, *trnL-CAA*(× 2), *trnL-UAA, *trnL-UAG*, *trnM-CAU*, *trnN-GUU*(× 2), *trnP-UGG*, *trnQ-UUG*, *trnR-ACG*(× 2), *trnR-UCU*, *trnS-GCU*, *trnS-GGA*, *trnS-UGA*, *trnT-GGU*, *trnT-UGU*, *trnV-GAC*(× 2), *trnV-UAC, *trnW-CCA*, *trnY-GUA*
Photosynthesis	Subunits of ATP synthase	*atpA*, *atpB*, *atpE*, *atpF, *atpH*, *atpI*
	Subunits of NADH-dehydrogenase	*ndhA, *ndhB(×2), *ndhC*, *ndhD*, *ndhE*, *ndhF*, *ndhG*, *ndhH*, *ndhI*, *ndhJ*, *ndhK*
	Subunits of cytochrome b/f complex	*petA*, *petB, *petD*, *petG*, *petL*, *petN*
	Subunits of photosystem I	*psaA*, *psaB*, *psaC*, *psaI*, *psaJ*, *ycf4*
	Subunits of photosystem II	*psbA*, ②*psbB*, *psbC*, *psbD*, *psbE*, *psbF*, *psbH*, *psbI*, *psbJ*, *psbK*, *psbL*, *psbM*, *psbN*, *psbT*, ③*psbZ*
	Subunit of rubisco	*rbcL*
Other genes	LhbA	④*lhbA*
	Subunit of Acetyl-CoA-carboxylase	*accD*
	c-type cytochrom synthesis gene	*ccsA*
	Envelop membrane protein	*cemA*
	Protease	**clpP
	Translational initiation factor	*infA*
	Maturase	*matK*
Unknown function	Conserved open reading frames	*ycf1*, *ycf2*(×2), **ycf3, *ycf15*(×2)

**Note:** (×2): Two gene copies in IRs; *: gene containing a single intron; **: gene containing two introns; ①:One copy of *rpl2* gene is missing in the chloroplast genome of *P. peruviana*; ②:*psbB* gene is missing in the chloroplast genome of *P. peruviana*; ③:*psbZ* gene exists only in chloroplast of *P. minima*; ④: *lhbA* gene is missing in the chloroplast genome of *P. minima*

### Codon usage in *Physalis* cp genomes

After alignment of the five *Physalis* cp genomes in MEGA, a total of 20 amino acids were found encoded with differential usage depending on the *trnL* codons. Methionine and tryptophan only presented one *trnL* each. Whereas phenylalanine, tyrosine, histidine, glutamine, asparagine, lysine, aspartic acid, glutamic acid, and cysteine were encoded by two synonymous codons (Additional File 1: Table S3 and Additional File 2: Fig. S4).

### IR expansion and contraction

**Fig. 2:**
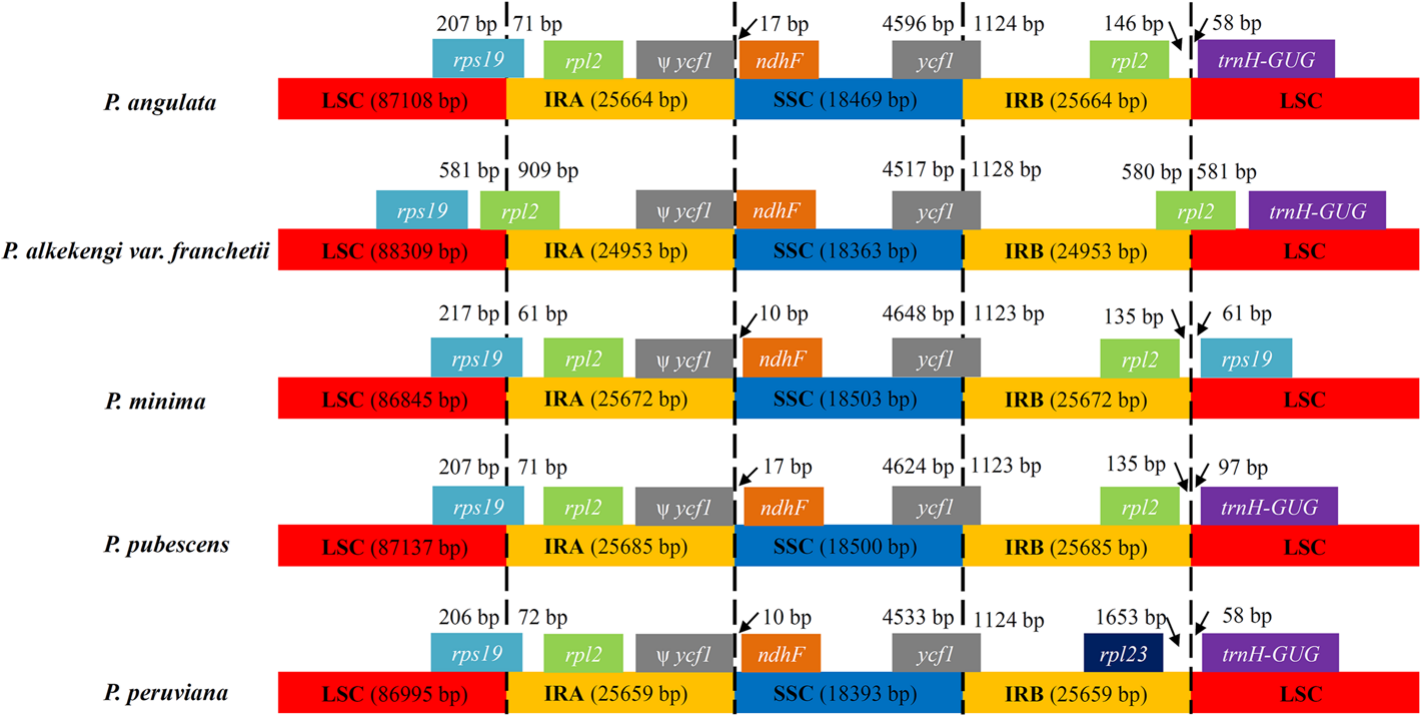
Comparisons of the borders of LSC, SSC, and IR regions among five *Physalis* chloroplast genomes

The IR regions (A and B) of the five *Physalis* cp genomes are the most conserved regions, being 24,953 to 25,685 bp in length. However, there are potential expansions and contractions of IR borders, which are considered to be evolutionary events and the main cause of cp genome length changes. The LSC/IR and SSC/IR borders of the *Physalis* cp genomes were compared (Fig. 2). The *rps19*, *rpl2*, *rpl23* and *trnH-GUG* genes were mainly distributed near the LSC/IR border, while *ycf1* and *ndhF* genes were distributed near the SSC/IR border. The gene *ycf1* crossed the SSC/IRB region, and the pseudogene fragment*ψycf1* was located at the IR-A region, near the SSC/IR-A border. Compared with the SSC/IR border, the LSC/IR border displayed a large variation. In *P. alkekengi* var. *franchetii*, the *rps19* gene was located completely in the LSC region. However, the *rps19* genes of *P. angulata*, *P. minima*, *P. pubescens*, and *P. peruviana* extended into the IRA region by 71, 61, 71, and 72 bp, respectively. There were two copies of the *rpl2* genes in *P. angulata*, *P. minima*, and *P. pubescens*, and they were located in the IR-A and IRB regions, near the LSC/IR borders. In *P. alkekengi* var. *franchetii*, the two copies of the *rpl2* gene span the LSC/IRA and LSC/IRB borders, respectively. One copy of the *rpl2* gene was missing at the LSC/IRB border in *P. peruviana*; instead, there was a *rpl23* gene at 1653 bp in the IRB region of the LSC/IRB border.

### Genomes sequence divergence among *Physalis* species

**Fig. 3:**
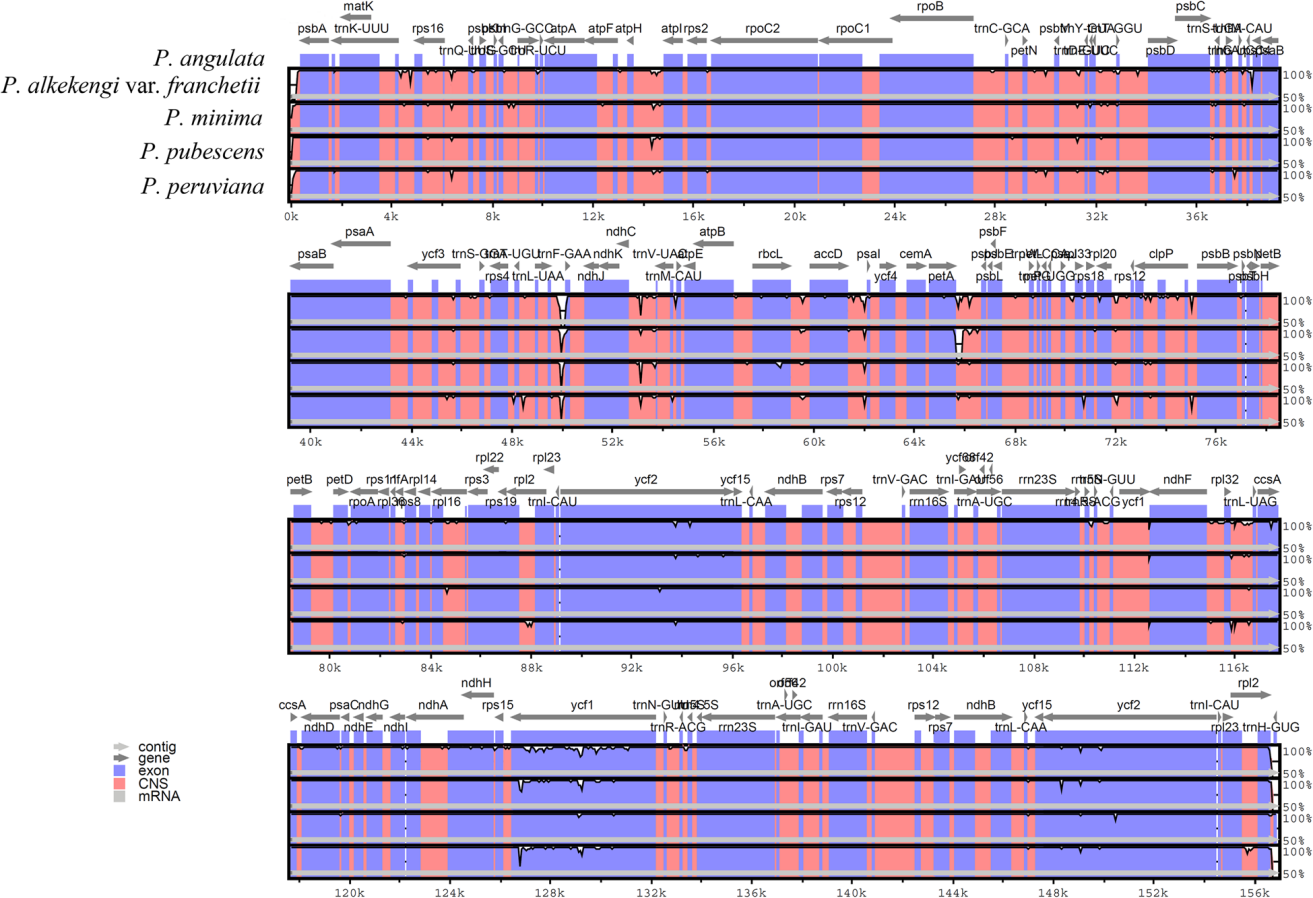
Comparative plots based on sequence identity of chloroplast genome of *Physalis* species, using *P. angulata* as the reference genome (upper plot). Plots were constructed with mVISTA software. Chloroplast coding regions are indicated in blue and non-coding regions in red, notice the reduction in sequence identity by reduction of the blue/red shadowing (white spaces)

The complete cp genomes of the five *Physalis* species were compared and plotted using mVISTA software by aligning the four cp genomes with the reference *P. angulata*, to elucidate the levels of sequence divergence (Fig. 3). LSC and SSC regions had higher sequence divergences than the IR regions. The sequence divergence in the coding region was limited, and most of the sequence divergence was concentrated in the non-coding region. At the genome level, the genetic distances among the five *Physalis* species ranged from 0.0007 to 0.0048, and the average genetic distance was just 0.0029 (Additional file 1: Table S4).

### Repeat sequences and SSR analysis

**Fig. 4:**
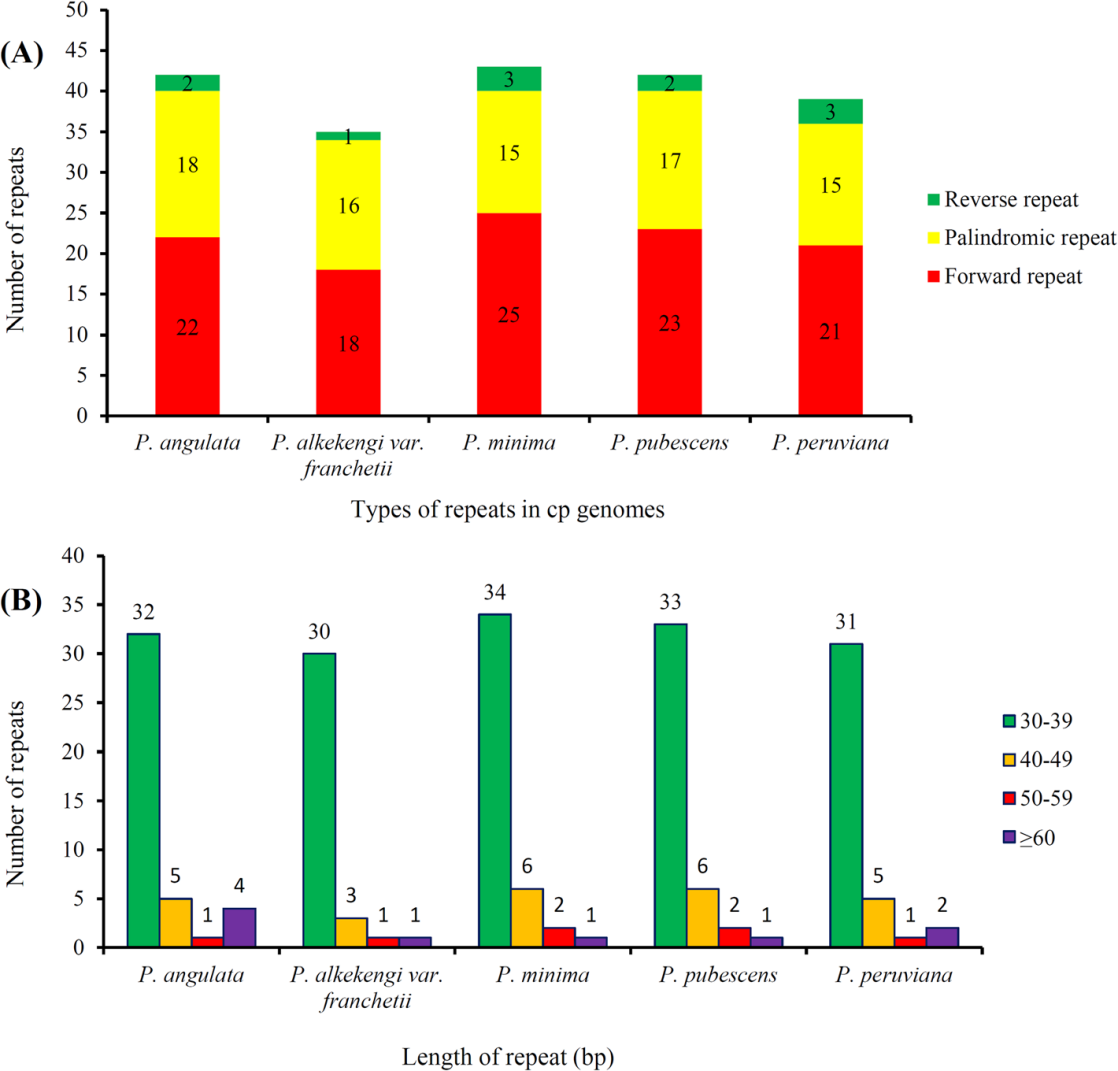
Repeated sequences in five *Physalis* chloroplast genomes. **a** Total of three repeat types in five *Physalis* chloroplast genomes; **b** Numbers of repeat sequences by length

REPuter was used to analyze the repeat sequences in each cp genome. A total of 201 repeat sequences were identified, including 109 forward repeats, 81 palindromic repeats, and 11 reverse repeats of at least 30 bp per repeat unit with a sequence identity ≥90% (Fig. 4). The distribution of repeats per genome, and length of repeat and number of such repeated sequences per species are shown in Fig. 4 a and b, respectively.

**Fig. 5:**
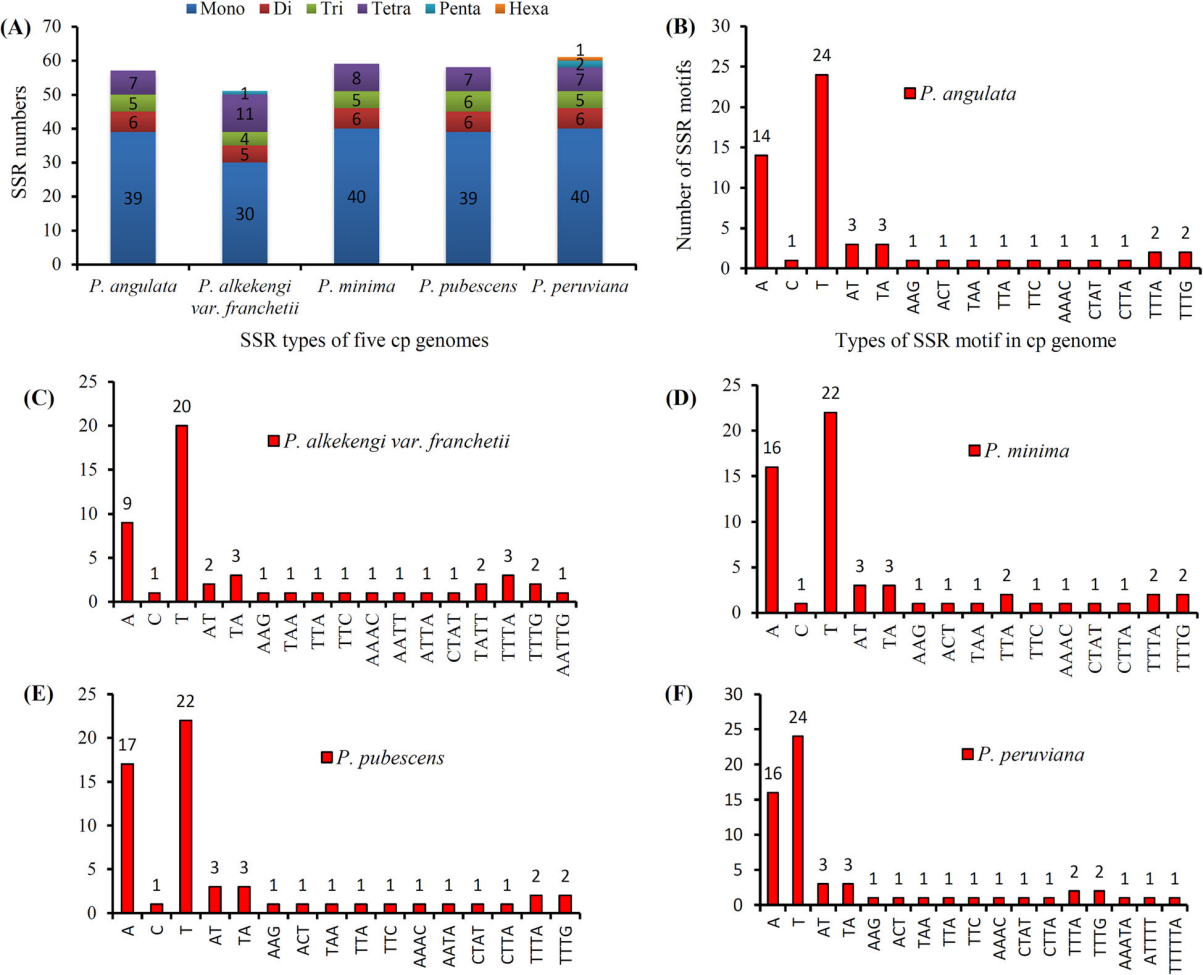
SSR loci analysis of five *Physalis* chloroplast genomes. **a** Numbers of different SSRs types detected in the five genomes; **b**–**e**: Frequency rates of identified SSR motifs in different repeat class types

The SSRs, which usually consist of a series repeat units of 1–6 bp in length (labelled as mono- to hexa- mer in Fig. 5a), were distributed throughout the genome. In total 286 SSRs, with lengths of at least 10 bp, were detected, with a distribution ranging from 51 to 61 SSRs per genome (Fig. 5a). The majority of these SSRs were mononucleotides (poly-A or poly-T mainly), with 30–40 members in each cp genome (Fig. 5b–f). Only dinucleotides AT or TA were found in all species, and the sole hexanucleotide (TTTTTA) was detected only in *P. peruviana* (Fig. 5f). Trinucleotides (AAG, ACT, TAA, TTA, and/or TTC), tetranucleotides (AAAC, AATA, CTAT, CTTA, TTTA, and/or TTTG) and pentanucleotide SSRs (AATTG and/or AAATA), were found with a specific distribution, that may be used for future population studies (Fig. 5b to f).

### Phylogenetic analysis

**Fig. 6:**
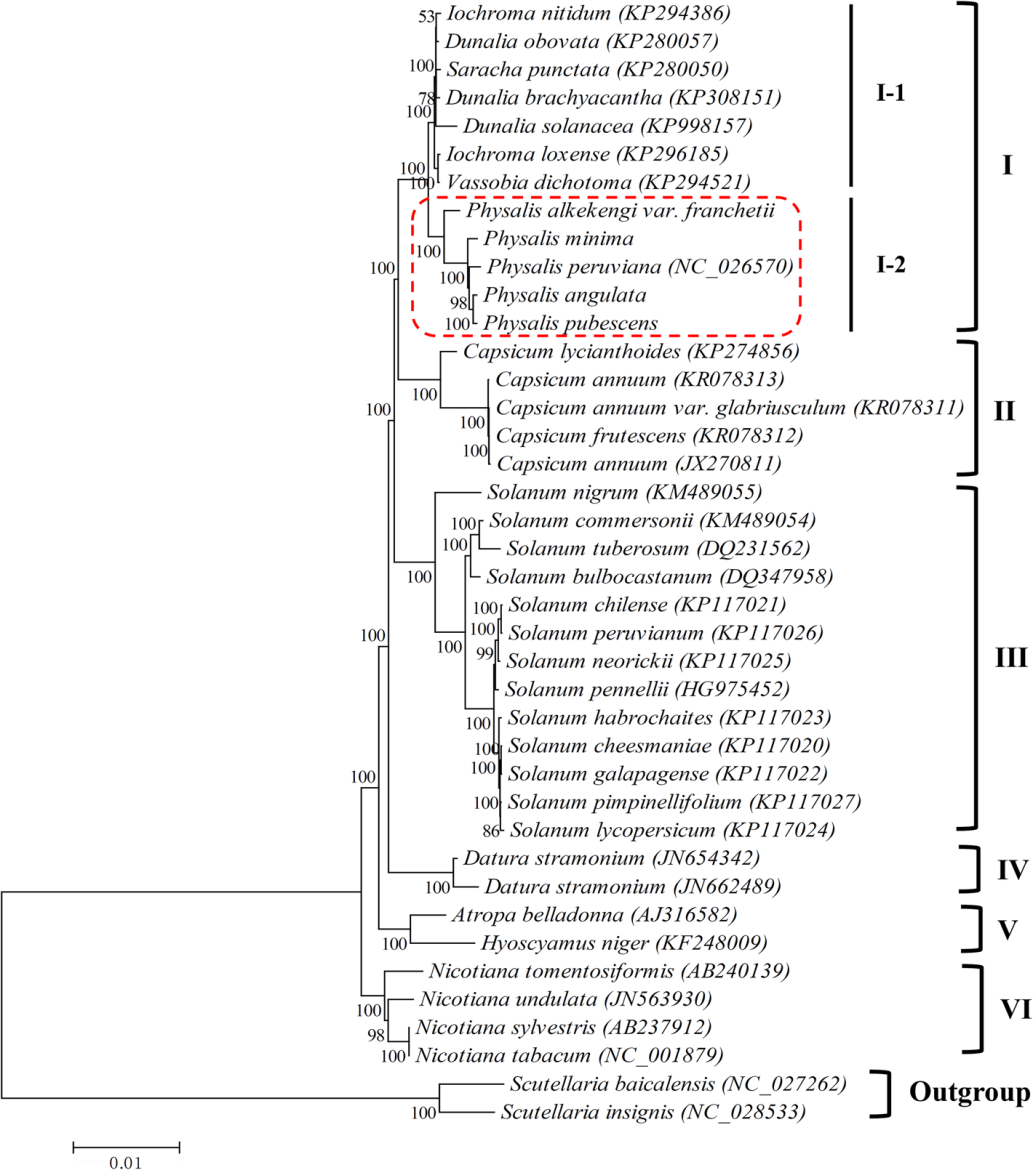
Maximum-likelihood (ML) tree based on the complete chloroplast genome sequences for 36 species of Solanaceae. Numbers above branches indicate bootstrap support, and circled by the red dotted lines are the five *Physalis* species

To examine the phylogenetic positions of the five *Physalis* species and their relationships within Solanaceae, ML and NJ phylogenetically analyses were performed using 38 complete cp genomes from 36 species belonging to 11 genera of Solanaceae. Phylogenetic reconstruction by ML and NJ (Fig. 6 and Additional File 2: Fig. S5) divided all species into six groups (I to VI), with slightly differences based bootstrap support (BS) values for each tree topology. Group I was the most complex, with 12 species, and was further divided into two subgroups. One subgroup containing the five species *Physalis* species studied in this work (I-2, BS = 100%), with *P. alkekengi* var. *franchetii* as basal species. And the second sub-group (I-1, BS = 100%) with species from *Iochroma*, *Dunalia*, *Saracha*, and *Vassobia* genera. Group II contained four *Capsicum* species (*C. annuum*, *C. annuum* var. *glabriusculum*, *C. lycianthoides* and *C. frutescens*) in both the ML and NJ phylogenetic trees with 100% bootstrap values. Group III included 13 *Solanum* species (BS = 100% for both ML and NJ trees). *Datura stramonium* clustered into Group IV in both ML and NJ phylogenetic trees with 100% bootstrap values. Group V included *Atropa belladonna* and *Hyoscyamus niger*. The four *Nicotiana* species, *N. sylvestris*, *N. tomentosiformis*, *N. undulate*, and *N. tabacum*, were distant from any other Solanaceae species and were assigned into group VI (BS = 100% for both ML and NJ trees).

## Discussion

### Cp genome structure and sequence differences

In this study, four *Physalis* cp genomes were obtained using Illumina MiSeq and were compared with the published cp genome of *P. peruviana*. Illumina MiSeq is a next-generation sequencer that integrates amplification, sequencing, and data analysis on a single instrument, and was released by Illumina in 2011 [38]. Illumina MiSeq is quite closely matched in terms of utility and ease of workflow, and has good applications for the chloroplast genome sequencing [17, 39]. The comparative analysis of the five *Physalis* cp genomes showed highly conserved genes and structures. Like those of most angiosperms, the cp genomes of the five *Physalis* have a quadripartite structure that is typically composed of one LSC, one SSC, and two IR regions [15, 40]. The sizes of the cp genomes of *P. angulata*, *P. alkekengi* var. *franchetii*, *P. minima*, *P. pubescens*, and *P. peruviana* ranged from 156,578 bp to 157,007 bp, which suggested that the cp genome length in *Physalis* is highly conserved. The cp genomes of angiosperms evolve at relatively fast rates, and inversions and gene losses occur during the process of evolution [16]. In terms of gene composition, most of the coding genes, tRNAs and rRNAs of the five *Physalis* species are the same, but there are also slight differences. For example, the cp genome of *P. minima* has an additional *psbZ* gene, while the *lhbA* gene is missing, suggesting that gene deletion and insertion have occurred during the evolution of *P. minima*. In addition, the *psbB* gene and one copy of the *rps12* gene are missing in the cp genome of *P. peruviana*. In fact, in other plant cp genomes, there have been many reports of the loss of *lhbA*, *infA*, *rpl22* and *rps16*, as well as intron and copy deletions of *rpl2*, *clpP* and *rps12* [20, 41–43].

The cp genomes of land angiosperms are highly conserved, but the expansion and contraction of the IR and SC boundaries are believed to be the main reasons for changes in cp genome size [40, 44]. For example, inversions and/or gene loss events were identified in the cp genome of *Astragalus membranaceus* [16], and that of *Taxus chinensis* var. *mairei* was found to lack a copy of the IR region [25]. *Tetracentron* cp genomes showed expansion/contraction events in the IR region [45], and those of *Veroniceae* contained *rps19* gene duplications in the IR region [46]. After comparing the cp genomes among the five *Physalis* species, we found that the boundary region between the SSC and two IR regions was relatively conserved, and the distribution and specific locations of gene types in this region were highly consistent. Compared with the other four *Physalis* species, the IR region of *P. alkekengi* var. *franchetii* showed shrinkage, and its length was the smallest (24,953 bp), mainly because the *rpl2* gene located at the LSC/IR boundary expanded the LSC region by 581 bp. In *P. peruviana*, there was no *rpl2* gene at the LSC/IRB boundary, unlike in the other four *Physalis* species. Additionally, only the *rps19* gene of *P. minima* was found at the LSC/IRA and LSC/IRB boundaries of the five plants, indicating that it was replicated. This phenomenon also found in the cp genome of *Veroniceae nakaiana* [46]. Therefore, changes in the LSC/IRB boundary appear to be the main contributors to the expansion/contraction of IR regions in *Physalis*.

Codon usage is a key factor in expressing genetic information correctly [47]. All five *Physalis* species shared the same codon usage, including 61 amino acid codons (containing one initiation codon, AUG) and three termination codons (UAA, UAG, and UGA). There were differences in the number and types of codons encoding 20 amino acids, and there was preferential codon usage. Most of the preferred amino acid-encoding codons had A or U as the third nucleotide. This phenomenon has been found in many angiosperms, such as *Aconitum barbatum* var. *Puberulum* [47]*, Nicotiana otophora* [48] and *Oryza minuta* [49]. The codon usage frequency was different in other cp genomes, which might be related to the hydrophilicity, synonymy substitution rate, and/or expression level of the codon [50]. Codon preference is closely correlated with the evolutionary pattern of the species. Therefore, the study of codon use is of great value to further understand the historical evolution of the genus *Physalis*.

In most higher plants, there is less variation in the IR regions than in the SC regions, and this is mainly caused by repeated corrections caused by gene transformations between the two IR regions [40]. The mVISTA results showed that the cp genomes of *Physalis* had a low degree of sequence divergence, and the conservation of the IR regions was higher than that of the SC region. In addition, the conservation of coding region was higher than that of non-coding region, which was consistent with most cp genomes of higher angiosperms [40].

### Repeat sequences and SSR sites

Repeat sequences are useful in phylogenetic studies and play crucial roles in genome recombination [48]. Furthermore, comparative studies of different cp genomes have shown that repeated sequences are important factors causing gene insertion, deletion, and replacement [51, 52]. A repeat analysis of the five *Physalis* cp genomes detected 201 repeat sequences, most of which are 30–39 bp in length. Among the five *Physalis* species, *P. minima* has the largest number of repeated sequences. Genome recombination and sequence variation are mainly caused by slipped-strand mismatches and inappropriate recombinations of repeated sequences [48, 51]. These repeats are the basis of genetic markers for population and phylogenetic studies, being widely used because of their high polymorphism rates among other characteristics [53–56]. In this study, 286 SSR loci were detected, most of them of A/T type as previously reported [56, 57].

### Phylogenetic analysis

Owing to the large number of species, similar morphology, and wide distribution areas, *Physalis* plants are considered to be a relatively complex taxonomic group at both the morphological and molecular levels. Whitson and Manos (2005) used the ITS sequence and *Waxy* gene to conduct phylogenetic studies on the genus *Physalis* and its relatives [1]. Many morphological characteristics of *Physalis* appear to be homoplasious, and several previously defined intrageneric taxa of *Physalis* are not monophyletic [1]. Olmstead et al. (2008) presented a phylogenic study of Solanaceae, which included the five *Physalis* species *P. heterophylla*, *P. peruviana*, *P. philadelphica*, *P. alkekengi*, and *P. carpenter*, based on the cp DNA regions *ndhF* and *trnL-F* [32]. The study indicated that the genus *Physalis* is closely related to the genera *Margaranthus*, *Chamaesaracha*, *Quincula*, and *Oryctes*, and that *P. alkekengi* and *P. carpenteri* are not monogamous in evolution compared the other three *Physalis* species. In our previous studies in 2016 and 2018 [3, 33], the ITS2 sequence and cp *psbA*-*trnH* region, respectively, were used for the molecular identification and phylogenetic analysis of *Physalis* species. The conclusions were similar to those obtained by Whitson and Manos (2005) [1] and Olmstead et al. (2008) [32]. A systematic classification of *Physalis* species should be further explored. These studies have laid an important foundation for the classification and identification of *Physalis* species. However, the lengths of nuclear/cp gene sequence segments are relatively short, in which limits phylogenetic studies and results in phylogenetic trees that have low support values. Based on whole cp genome sequences, the present study conducted a phylogenetic analysis of 36 species in 11 genera (including the genus *Physalis*) of Solanaceae. ML and NJ analyses results showed that the tested *Physalis* species formed a single line in the phylogenetic evolution of Solanaceae (support rate of 100%) and are closely related to other genera, including *Iochroma*, *Dunalia*, *Saracha*, and *Vassobia*. *P. alkekengi* var. *franchetii* was distantly related to the other four *Physalis* species (support rate of 100%); therefore, we speculated that *P. alkekengi* var. *franchetii* differentiated earlier than the other four *Physalis* species during genetic evolution. To some extent, this result also supports the opinion that *P. alkekengi* var. *franchetii* should be classified into a small genus [1, 3]. Of course, only partial cp genomic sequences of *Physalis* and Solanaceae plants are available at present; therefore, the systematic classification of *Physalis* species cannot be completed. We plan to obtain more cp genomes of *Physalis* species using high-throughput sequencing in the future, which will allow us to more accurately analyze the phylogenetic relationships among *Physalis* species.

Although many studies have shown that the use of cp genomes has advantages in phylogenetic studies, there are still many problems [28]. For example, different species have different evolutionary rates, and for some groups with rapid evolutionary rates, using the whole cp genome information alone cannot completely determine their phylogenetic evolution [58]. In addition, cp DNA is parthenogenetic, and its genomic information can only reflect the evolutionary process of the maternal or paternal line, but it cannot be used to completely interpret the whole systematic evolution of the species itself [59]. Therefore, to better reveal the phylogenetic evolution of *Physalis* species, in addition to studies of the cp genomes, future studies should be combined with data analyses of nuclear and mitochondrial genomes.

## Conclusions

In this study, the cp genomes of four *Physalis* species, *P. angulata*, *P. alkekengi* var. *franchetii*, *P. pubescens*, and *P. minima* were first obtained through high-throughput sequencing. The comparative genomic analysis performed, which included the published cp genome of *P. peruviana,* allowed us to determine the circular nature with the typical quadripartite structure of the *Physalis* cp genome. The whole *Physalis* cp genomes were relatively conserved, with differences at the boundaries IR/SC and LSC/IR. Nearly 290 SSR loci have been identified, which can be used as molecular markers in a future *Physalis* intraspecific diversity study. Whole cp genome allowed to reconstruct the phylogenetic trees of Solanaceae, identifying six group of species, and finding *Physalis* as an independent clade within Solanaceae group I. Our results enrich the data on the cp genomes of the genus *Physalis* and lay an important foundation for the accurate molecular identification and phylogenetic reconstruction of *Physalis* species.

## Methods

### Plant materials, DNA extraction and sequencing

Four species widely distributed in China, *P. angulata*, *P. alkekengi* var. *franchetii*, *P. minima* and *P. pubescens*, were field-collected (Details of sampling information of the four *Physalis* species collected in the study were shown in Additional File 1: Table S1). The formal identification of the plant material was undertaken by Dr. Huizhong Wang (Hangzhou Normal University). Voucher specimens of all the collected species were deposited at the Zhejiang Provincial Key Laboratory for Genetic Improvement and Quality Control of Medicinal Plants, Hangzhou Normal University (Additional File 1: Table S1). Permission was not necessary for collecting these species, which have not been included in the list of national key protected plants. Clean, healthy, fresh green leaves from the collected *Physalis* plants were sampled (6 specimens per each species). Leaves were surface washed, dried and stored at − 80 °C till DNA extraction.

Total genomic DNA was extracted from ~ 0.1 g of preserved leaves (mix of equal amounts of 6 individuals) according to a modified CTAB method [60]. The modification was mainly in the CTAB extraction buffer which contained 4% CTAB instead of 2%, ~ 0.2% DL-dithiothreitol (DTT) and 1% polyvinyl poly-pyrrolidone (PVP), the rest of the protocol was as described [60]. Complete cp genome of each species was obtained by Long-range PCR on total genomic DNA, as in previous works [17, 61]. Briefly, the PCR was carried out in 25 μL containing 1 × PrimeSTAR GXL buffer [10 mM Tris-HCl (pH 8.2), 1 mM MgCl_2_, 20 mM NaCl, 0.02 mM EDTA, 0.02 mM DDT, 0.02% Tween 20, 0.02% NP-40, and 10% glycerol], 1.6 mM dNTPs, 0.5 μM of each primer pair (as described in Yang et al. [61]) (Additional File 1: Table S5), 1 U PrimeSTAR GXL DNA polymerase (TaKaRa BIO INC.; Dalian, China), and 50 ng genomic DNA template. The PCR was performed using a GeneAmp PCR System 9700 DNA Thermal Cycler (PerkinElmer, Norwalk, CT, USA) with the following PCR program: 94 °C for 1 min, followed by 30 cycles 68 °C for 15 min, and a final extension at 72 °C for 10 min. Nine PCR reactions were performed for each *Physalis* species. The PCR products from the above reactions were then mixed in roughly equal proportions for Illumina MiSeq sequencing. These mixtures were fragmented and used for short insert (500 bp) library construction, following the manufacturer’s protocol (Illumina) [17]. DNA libraries of different species were run on an Illumina Miseq machine with paired-end, 250 bp reads at the Germplasm Bank of Wild Species in Southwest China, Kunming Institution of Botany, Chinese Academy of Sciences.

### Genome assembly, annotation and comparative analysis

De novo and reference-guided strategies were used to assemble cp genomes. First, Illumina short reads were assembled into contigs using NGS QC Toolkit v2.3.3 (www.nipgr.res.in/ngsqctoolkit.html). Second, the high quality pair-ended reads were assembled using CLC Genomics Workbench version 8 (CLC Bio, Aarhus, Denmark) and SOAPdenovo (http://soap.genomics.org.cn/soapdenovo.html) with a k-mer length of 63. Third, highly similar genome sequences were identified using BLAST (http://blast.ncbi.nlm.nih.gov/) with default parameters. Output scaffolds/contigs larger than 1000 bps were mapped to the reference cp genome of *P. peruviana* (NC_026570). Finally, we determined the order of aligned scaffolds/contigs according to the reference genome and resolved any gaps that were present by mapping the raw reads to the assembly.

The Dual Organellar GenoMe Annotator (DOGMA) (http://dogma.ccbb.utexas.edu/) tool [62] was used to annotate the four complete *Physalis* cp genomes. Start and stop codons of protein-coding genes and intron positions were manually corrected based on the reference genome (NC_026570). DOGMA and tRNA scan-SE version 1.21 [63] were used to obtain and identify tRNA genes. The circular gene maps were constructed using the OrganellarGenomeDRAW tool followed by manual modification [64]. The cp genomes after annotation were submitted to the GenBank database (GenBank accession numbers: MH045574, MH045575, MH045576 and MH045577). Cp genome comparisons among the five *Physalis* species were performed using the mVISTA program (http://genome.lbl.gov/vista/mvista/about.shtml). MEGA 6 software was used to analyze GC content, codon usage and phylogenetic analyses as described below [65].

### Repeat sequences and SSR analysis

The Perl script MISA (http://pgrc.ipk-gatersleben.de/misa/) [66] was used to detect potential microsatellites (SSRs) in the *Physalis* cp genomes. The parameters were set as follows: 10 repeat units for mononucleotide SSRs, 5 repeat units for dinucleotide SSRs, 4 repeat units for trinucleotide SSRs, and 3 repeat units for tetra-, penta- and hexanucleotide repeats. REPuter was used to identify forward (direct), reverse, and palindromic repeats, within the cp genome, with a minimum repeat size of 30 bp and 90% sequence identity (Hamming distance of 3) [67].

### Phylogenetic analysis

To elucidate the phylogenetic positions of *Physalis* species within the Solanaceae family, multiple alignments were performed using the complete cp genome sequences of 36 Solanaceae species representing 11 genera (Additional File 1: Table S6), including *Scutellaria baicalensis* (NC_027262) and *S. insignis* (NC_028533), as outgroups. The MAFFT7.017 and ClustalX alignment software were used to compare and analyze the complete cp genome sequences of all the species, manual adjustments were made where necessary [68]. Maximum-likelihood (ML) and neighbor-joining (NJ) analyses were performed using MEGA 6 [65], using the general time reversible model with substitution-rate among sites of gamma distribution with invariant sites (GTR + G + I), with complete gap elimination and 1000 bootstrap repeats to ascertain branch support, as implemented in MEGA. Nucleotide and phylogeny inference models were selected after model testing in MEGA.

## Supplementary information

**Additional file 1: Table S1.** Information on the four *Physalis* species used in the study. **Table S2.** Quality control of the Illumina sequencing of chloroplast genome of *Physalis* species. **Table S3.** Relative synonymous codon usage (RSCU) in five *Physalis* chloroplast genomes. **Table S4.** Evolutionary divergence among *Physalis* species based on complete chloroplast genome sequences. **Table S5.** Universal primers for amplifying complete chloroplast genomes. **Table S6.** The 36 studied species belonging to 11 genera of Solanaceae, and the corresponding chloroplast whole genome GenBank accession number.

**Additional file 2: Figure S1.** Gene map of the *P. alkekengi* var. *franchetii* chloroplast genome. Genes shown outside the outer circle are transcribed clockwise, and those inside are transcribed counterclockwise. Genes belonging to different functional groups are color coded. The darker gray in the inner circle indicates the GC content, and the lighter gray indicates the AT content. The inner circle also indicates that the chloroplast genome contains two copies of the inverted repeat (IRA and IRB), a large single-copy region (LSC) and a small single-copy region (SSC). The map was constructed using OrganellarGenomeDRAW. **Figure S2.** Gene map of the *P. minima* chloroplast genome. Genes shown outside the outer circle are transcribed clockwise, and those inside are transcribed counterclockwise. Genes belonging to different functional groups are color coded. The darker gray in the inner circle indicates the GC content, and the lighter gray indicates the AT content. The inner circle also indicates that the chloroplast genome contains two copies of the inverted repeat (IRA and IRB), a large single-copy region (LSC) and a small single-copy region (SSC). The map was constructed using OrganellarGenomeDRAW. **Figure S3.** Gene map of the *P. pubescens* chloroplast genome. Genes shown outside the outer circle are transcribed clockwise, and those inside are transcribed counterclockwise. Genes belonging to different functional groups are color coded. The darker gray in the inner circle indicates the GC content, and the lighter gray indicates the AT content. The inner circle also indicates that the chloroplast genome contains two copies of the inverted repeat (IRA and IRB), a large single-copy region (LSC) and a small single-copy region (SSC). The map was constructed using OrganellarGenomeDRAW. **Figure S4.** Amino acid frequencies in the chloroplast genomes of five *Physalis* species. **Figure S5.** Neighbor-joining (NJ) tree based on the complete chloroplast genome sequences of 36 species of Solanaceae. Numbers above branches indicate bootstrap support, and circled by the red dotted lines are the five *Physalis* species.

## Data Availability

The complete chloroplast genomes of *P. angulata*, *P. alkekengi* var. *franchetii*, *P. minima* and *P. pubescens* were submitted to the NCBI database (https://www.ncbi.nlm.nih.gov/) with GenBank accession numbers MH045574 (*P. angulata*), MH045575 (*P. alkekengi* var. *franchetii*), MH045577 (*P. minima*) and MH045576 (*P. pubescens*). All other data and material generated in this manuscript are available from the corresponding author upon reasonable request.

## Abbreviations

Cp: Chloroplast
ITS: Internal transcribed spacer
SSR: Simple sequence repeat
IRs: Inverted repeats
LSC: Large single-copy
SSC: Small single-copy
ML: Maximum-likelihood
NJ: Neighbor-joining
BS: Branch support
