# Supplementary material for: Complete chloroplast genomes of four Physalis species (Solanaceae): lights into genome structure, comparative analysis, and phylogenetic relationships
Source: BMC Plant Biol. 2020 May 28;20:242. doi: 10.1186/s12870-020-02429-w (PMC7254759; doi:10.1186/s12870-020-02429-w)
Supplement: Supplementary file 1 — Additional file 1: Table S1. Information on the four Physalis species used in the study. Table S2. Quality control of the Illumina sequencing of chloroplast genome of Physalis species. Table S3. Relative synonymous codon usage (RSCU) in five Physalis chloroplast genomes. Table S4. Evolutionary divergence among Physalis species based on complete chloroplast genome sequences. Table S5. Universal primers for amplifying complete chloroplast genomes. Table S6. The 36 studied species belonging to 11 genera of Solanaceae, and the corresponding chloroplast whole genome GenBank accession number. [file 12870_2020_2429_MOESM1_ESM.docx]

**BMC Plant Biology**

**SUPPLEMENTARY INFORMATION FOR:**

**Complete chloroplast genomes of four *Physalis* species (Solanaceae): lights into genome structure, comparative analysis, and phylogenetic relationships**

Shangguo Feng^1, 2^, Kaixin Zheng^1, 2^, Kaili Jiao^1, 2^, Yuchen Cai^1, 2^, Chuanlan Chen ^1^, Yanyan Mao^1^, Lingyan Wang^1^, Xiaori Zhan^1, 2^, Qicai Ying^1, 2^ and Huizhong Wang^1, 2^ *

This file contains the supplementary information corresponding to tables, identified as such in the main manuscript. The order of the tables is kept as their first citation in the main text.

**Table S1.** Information on the four *Physalis* species used in the study

| Species | Voucher No. | Locality information | Longitude | Latitude | Altitude (m) |
| --- | --- | --- | --- | --- | --- |
| *Physalis* *angulata* L. | PHZ0001 | Xiaoshan, Hangzhou, Zhejiang, China | 120°15′ | 30°11′ | 8 |
| *P. alkekengi* var. *franchetii* (Mast.) Makino | PHZ4001 | Nong’an, Changchun, Jilin, China | 125°10′ | 44°25′ | 196 |
| *P. minima* L. | PHZ3003 | Mudan, Heze, Shandong, China | 115°24′ | 35°15′ | 53 |
| *P. pubescens* L. | PHZ2001 | Faku, Shenyang, Liaoning, China | 123°24′ | 42°30′ | 144 |

**Table S2.** Quality control of the Illumina sequencing of chloroplast genome of *Physalis* species

| Sample name | Clean reads | Clean bases | Read length | Mean coverage | Q20 (%) |
| --- | --- | --- | --- | --- | --- |
| *P. angulata* | 181,340 | 43,753,304 | 250 bp | 480 | 90.09 |
| *P. alkekengi* var. *franchetii* | 628,082 | 150,385,353 | 250 bp | 1756 | 92.81 |
| *P. minima* | 878,611 | 189,007,226 | 250 bp | 1027 | 91.72 |
| *P. pubescens* | 367,318 | 85,487,467 | 250 bp | 946 | 93.57 |

**Table S3.** Relative synonymous codon usage (RSCU) in five *Physalis* chloroplast genomes

| Amino acid | Codon | *P. angulata* | | *P. alkekengi* var. *franchetii* | | *P. minima* | | *P. pubescens* | | *P. peruviana* | |
| --- | --- | --- | --- | --- | --- | --- | --- | --- | --- | --- | --- |
|  |  | Number | RSCU | Number | RSCU | Number | RSCU | Number | RSCU | Number | RSCU |
| Phe | UUU | 2271 | 1.24 | 2262 | 1.2 | 2259 | 1.23 | 2205 | 1.2 | 2245 | 1.23 |
|  | UUC | 1384 | 0.76 | 1493 | 0.8 | 1413 | 0.77 | 1456 | 0.8 | 1415 | 0.77 |
| Leu | UUA | 1210 | 1.33 | 991 | 1.16 | 1134 | 1.27 | 1212 | 1.35 | 1163 | 1.26 |
|  | UUG | 1101 | 1.21 | 1107 | 1.29 | 1065 | 1.19 | 1095 | 1.22 | 1078 | 1.17 |
|  | CUU | 1167 | 1.28 | 1093 | 1.28 | 1118 | 1.25 | 1145 | 1.28 | 1141 | 1.24 |
|  | CUC | 689 | 0.76 | 702 | 0.82 | 685 | 0.77 | 626 | 0.7 | 690 | 0.75 |
|  | CUA | 757 | 0.83 | 730 | 0.85 | 822 | 0.92 | 791 | 0.88 | 911 | 0.99 |
|  | CUG | 530 | 0.58 | 512 | 0.6 | 526 | 0.59 | 510 | 0.57 | 536 | 0.58 |
| Ile | AUU | 1818 | 1.24 | 1915 | 1.28 | 1820 | 1.26 | 1781 | 1.24 | 1740 | 1.22 |
|  | AUC | 1116 | 0.76 | 1171 | 0.78 | 1105 | 0.77 | 1071 | 0.74 | 1071 | 0.75 |
|  | AUA | 1450 | 0.99 | 1399 | 0.94 | 1400 | 0.97 | 1472 | 1.02 | 1485 | 1.04 |
| Met | AUG | 819 | 1 | 804 | 1 | 853 | 1 | 840 | 1 | 850 | 1 |
| Val | GUU | 840 | 1.38 | 758 | 1.32 | 857 | 1.47 | 818 | 1.44 | 805 | 1.36 |
|  | GUC | 417 | 0.69 | 455 | 0.79 | 426 | 0.73 | 402 | 0.71 | 417 | 0.71 |
|  | GUA | 758 | 1.25 | 694 | 1.21 | 651 | 1.11 | 682 | 1.2 | 686 | 1.16 |
|  | GUG | 419 | 0.69 | 386 | 0.67 | 404 | 0.69 | 370 | 0.65 | 452 | 0.77 |
| Pro | CCU | 755 | 1.15 | 643 | 1.06 | 679 | 1.1 | 685 | 1.12 | 702 | 1.12 |
|  | CCC | 637 | 0.97 | 629 | 1.03 | 625 | 1.01 | 624 | 1.02 | 630 | 1 |
|  | CCA | 827 | 1.26 | 774 | 1.27 | 768 | 1.24 | 732 | 1.2 | 761 | 1.21 |
|  | CCG | 414 | 0.63 | 386 | 0.63 | 403 | 0.65 | 406 | 0.66 | 425 | 0.68 |
| Thr | ACU | 645 | 1.18 | 640 | 1.17 | 638 | 1.14 | 671 | 1.19 | 592 | 1.07 |
|  | ACC | 541 | 0.99 | 565 | 1.03 | 587 | 1.05 | 562 | 1 | 564 | 1.02 |
|  | ACA | 659 | 1.2 | 605 | 1.11 | 653 | 1.16 | 666 | 1.18 | 701 | 1.27 |
|  | ACG | 350 | 0.64 | 378 | 0.69 | 365 | 0.65 | 353 | 0.63 | 356 | 0.64 |
| Ala | GCU | 487 | 1.25 | 469 | 1.25 | 438 | 1.2 | 504 | 1.3 | 472 | 1.24 |
|  | GCC | 340 | 0.87 | 375 | 1 | 360 | 0.99 | 382 | 0.99 | 384 | 1.01 |
|  | GCA | 484 | 1.24 | 406 | 1.08 | 420 | 1.15 | 412 | 1.07 | 439 | 1.15 |
|  | GCG | 250 | 0.64 | 256 | 0.68 | 237 | 0.65 | 249 | 0.64 | 233 | 0.61 |
| Tyr | UAU | 1410 | 1.34 | 1422 | 1.35 | 1455 | 1.34 | 1386 | 1.35 | 1462 | 1.37 |
|  | UAC | 696 | 0.66 | 685 | 0.65 | 717 | 0.66 | 664 | 0.65 | 678 | 0.63 |
| His | CAU | 905 | 1.4 | 937 | 1.39 | 977 | 1.43 | 950 | 1.41 | 959 | 1.39 |
|  | CAC | 391 | 0.6 | 409 | 0.61 | 390 | 0.57 | 394 | 0.59 | 422 | 0.61 |
| Gln | CAA | 1105 | 1.37 | 1084 | 1.39 | 1103 | 1.41 | 1080 | 1.34 | 1076 | 1.38 |
|  | CAG | 509 | 0.63 | 479 | 0.61 | 467 | 0.59 | 530 | 0.66 | 489 | 0.62 |
| Asn | AAU | 1743 | 1.4 | 1792 | 1.37 | 1727 | 1.39 | 1893 | 1.4 | 1765 | 1.38 |
|  | AAC | 750 | 0.6 | 828 | 0.63 | 754 | 0.61 | 802 | 0.6 | 785 | 0.62 |
| Lys | AAA | 2171 | 1.35 | 2078 | 1.35 | 2072 | 1.33 | 2089 | 1.34 | 2054 | 1.3 |
|  | AAG | 1047 | 0.65 | 998 | 0.65 | 1052 | 0.67 | 1024 | 0.66 | 1100 | 0.7 |
| Asp | GAU | 1125 | 1.44 | 1007 | 1.39 | 1027 | 1.41 | 1124 | 1.41 | 1079 | 1.43 |
|  | GAC | 434 | 0.56 | 447 | 0.61 | 429 | 0.59 | 465 | 0.59 | 434 | 0.57 |
| Glu | GAA | 1399 | 1.37 | 1331 | 1.39 | 1348 | 1.39 | 1342 | 1.36 | 1306 | 1.33 |
|  | GAG | 636 | 0.63 | 588 | 0.61 | 595 | 0.61 | 628 | 0.64 | 653 | 0.67 |
| Cys | UGU | 695 | 1.24 | 704 | 1.21 | 691 | 1.23 | 692 | 1.21 | 727 | 1.27 |
|  | UGC | 428 | 0.76 | 463 | 0.79 | 431 | 0.77 | 451 | 0.79 | 419 | 0.73 |
| Trp | UGG | 680 | 1 | 719 | 1 | 707 | 1 | 704 | 1 | 732 | 1 |
| Arg | CGU | 399 | 0.72 | 390 | 0.69 | 386 | 0.68 | 391 | 0.7 | 350 | 0.65 |
|  | CGC | 238 | 0.43 | 240 | 0.42 | 237 | 0.42 | 250 | 0.45 | 217 | 0.4 |
|  | CGA | 568 | 1.03 | 606 | 1.07 | 611 | 1.08 | 573 | 1.02 | 577 | 1.07 |
|  | CGG | 429 | 0.77 | 388 | 0.68 | 413 | 0.73 | 413 | 0.74 | 423 | 0.78 |
|  | AGA | 1069 | 1.93 | 1142 | 2.01 | 1110 | 1.97 | 1094 | 1.95 | 1060 | 1.96 |
|  | AGG | 621 | 1.12 | 642 | 1.13 | 630 | 1.12 | 642 | 1.15 | 622 | 1.15 |
| Ser | UCU | 1151 | 1.5 | 1138 | 1.41 | 1138 | 1.42 | 1222 | 1.5 | 1093 | 1.41 |
|  | UCC | 848 | 1.11 | 904 | 1.12 | 905 | 1.13 | 903 | 1.11 | 888 | 1.15 |
|  | UCA | 943 | 1.23 | 865 | 1.07 | 918 | 1.15 | 957 | 1.18 | 959 | 1.24 |
|  | UCG | 579 | 0.76 | 639 | 0.79 | 605 | 0.75 | 625 | 0.77 | 618 | 0.8 |
|  | AGU | 631 | 0.82 | 719 | 0.89 | 711 | 0.89 | 698 | 0.86 | 661 | 0.85 |
|  | AGC | 443 | 0.58 | 570 | 0.71 | 532 | 0.66 | 473 | 0.58 | 426 | 0.55 |
| Gly | GGU | 586 | 1.05 | 559 | 0.98 | 559 | 0.99 | 579 | 1.02 | 543 | 1 |
|  | GGC | 343 | 0.61 | 358 | 0.63 | 367 | 0.65 | 381 | 0.67 | 336 | 0.62 |
|  | GGA | 754 | 1.35 | 797 | 1.4 | 780 | 1.38 | 764 | 1.34 | 740 | 1.36 |
|  | GGG | 552 | 0.99 | 561 | 0.99 | 550 | 0.98 | 555 | 0.97 | 552 | 1.02 |
| Stop | UAA | 1142 | 1.19 | 1263 | 1.22 | 1255 | 1.2 | 1169 | 1.22 | 1200 | 1.17 |
| Stop | UAG | 788 | 0.82 | 806 | 0.78 | 851 | 0.82 | 758 | 0.79 | 890 | 0.87 |
| Stop | UGA | 958 | 1 | 1036 | 1 | 1019 | 0.98 | 948 | 0.99 | 996 | 0.97 |

**Table S4.** Evolutionary divergence among *Physalis* species based on complete chloroplast genome sequences

| species name | *P. angulata* | *P. alkekengi* var. *franchetii* | *P. minima* | *P. pubescens* | *P. peruviana* |
| --- | --- | --- | --- | --- | --- |
| *P. angulata* | 0.0000 |  |  |  |  |
| *P. alkekengi* var. *franchetii* | 0.0044 | 0.0000 |  |  |  |
| *P. minima* | 0.0017 | 0.0045 | 0.0000 |  |  |
| *P. pubescens* | 0.0007 | 0.0045 | 0.0017 | 0.0000 |  |
| *P. peruviana* | 0.0017 | 0.0048 | 0.0021 | 0.0018 | 0.0000 |

**Table S5.** Universal primers for amplifying complete chloroplast genomes

| Primer Name | Forward primer (5'-3') | Reverse primer (5'-3') |
| --- | --- | --- |
| CP_1_*trnI* | GGCTGAATGGTTAAAGCGCCCA | TTGCGTCCAATAGGATTTGAACCTATACC |
| CP_2_*trnG* | GGTTCGATTCCCGCTACCCGC | TGGTTCAAATCCAGCTCGGCCC |
| CP_3_*trnC* | CCCCGGTTCAAATCTGGGTGTCG | CGCCTTGAACCACTCGGCCA |
| CP_4_*trnS* | TGTAGGAGAGATGGCCGAGTGG | CCATTGCAATTGCCGGAAATACTAGGC |
| CP_5_*trnT* | ACGGCGGGAGTCATTGGTTCA | AGTTCGGTAGAACGTGGGTCTCCA |
| CP_6_*trnW* | TGAACCTACGACATCGGGTTTTGGAGA | ATGTACGAGGATCCCCGCTAAGCATC |
| CP_7_*petB* | GCTTGAGCTGTACGAGATGAAAGTCT | AGAGCGTGGAGGTTCGAGTCC |
| CP_8_*trnL* | GGACTCGAACCTCCACGCTCT | GCCGCTACTCGGACTCGAACC |
| CP_9_*trnL* | GGTTCGAGTCCGAGTAGCGGC | ACAGCCGACCGCTCTACCAC |

**Table S6**. The 36 studied species belonging to 11 genera of Solanaceae, and the corresponding chloroplast whole genome GenBank accession number

| Genus | Species | GenBank No. | Genus | Species | GenBank No. |
| --- | --- | --- | --- | --- | --- |
| *Physalis* | *Physalis angulata* | MH045574 | *Capsicum* | *Capsicum annuum* | KR078313; JX270811 |
|  | *P. alkekengi* var. *franchetii* | MH045575 |  | *C. annuum* var*. glabriusculum* | KR078311 |
|  | *P. minima* | MH045577 |  | *C. lycianthoides* | KP274856 |
|  | *P. pubescens* | MH045576 |  | *C. frutescens* | KR078312 |
|  | *P. peruviana* | KP295964 | *Vassobia* | *Vassobia dichotoma* | KP294521 |
| *Solanum* | *Solanum nigrum* | KM489055 | *Nicotiana* | *Nicotiana sylvestris* | AB237912 |
|  | *S. habrochaites* | KP117023 |  | *N. tomentosiformis* | AB240139 |
|  | *S. chilense* | KP117021 |  | *N. undulata* | JN563930 |
|  | *S. pennellii* | HG975452 |  | *N. tabacum* | NC_001879 |
|  | *S. bulbocastanum* | DQ347958 | *Atropa* | *Atropa belladonna* | AJ316582 |
|  | *S. cheesmaniae* | KP117020 | *Hyoscyamus* | *Hyoscyamus niger* | KF248009 |
|  | *S. pimpinellifolium* | KP117027 | *Dunalia* | *Dunalia solanacea* | KP998157 |
|  | *S. commersonii* | KM489054 |  | *D. brachyacantha* | KP308151 |
|  | *S. tuberosum* | DQ231562 |  | *D. obovata* | KP280057 |
|  | *S. neorickii* | KP117025 | *Iochroma* | *Iochroma nitidum* | KP294386 |
|  | *S. lycopersicum* | KP117024 |  | *I. loxense* | KP296185 |
|  | *S. peruvianum* | KP117026 | *Saracha* | *Saracha punctata* | KP280050 |
|  | *S. galapagense* | KP117022 | *Datura* | *Datura stramonium* | JN654342; JN662489 |
