# Supplementary material for: Complete chloroplast genomes of four Physalis species (Solanaceae): lights into genome structure, comparative analysis, and phylogenetic relationships
Source: BMC Plant Biol. 2020 May 28;20:242. doi: 10.1186/s12870-020-02429-w (PMC7254759; doi:10.1186/s12870-020-02429-w)
Supplement: Supplementary file 2 — Additional file 2: Figure S1. Gene map of the P. alkekengi var. franchetii chloroplast genome. Genes shown outside the outer circle are transcribed clockwise, and those inside are transcribed counterclockwise. Genes belonging to different functional groups are color coded. The darker gray in the inner circle indicates the GC content, and the lighter gray indicates the AT content. The inner circle also indicates that the chloroplast genome contains two copies of the inverted repeat (IRA and IRB), a large single-copy region (LSC) and a small single-copy region (SSC). The map was constructed using OrganellarGenomeDRAW. Figure S2. Gene map of the P. minima chloroplast genome. Genes shown outside the outer circle are transcribed clockwise, and those inside are transcribed counterclockwise. Genes belonging to different functional groups are color coded. The darker gray in the inner circle indicates the GC content, and the lighter gray indicates the AT content. The inner circle also indicates that the chloroplast genome contains two copies of the inverted repeat (IRA and IRB), a large single-copy region (LSC) and a small single-copy region (SSC). The map was constructed using OrganellarGenomeDRAW. Figure S3. Gene map of the P. pubescens chloroplast genome. Genes shown outside the outer circle are transcribed clockwise, and those inside are transcribed counterclockwise. Genes belonging to different functional groups are color coded. The darker gray in the inner circle indicates the GC content, and the lighter gray indicates the AT content. The inner circle also indicates that the chloroplast genome contains two copies of the inverted repeat (IRA and IRB), a large single-copy region (LSC) and a small single-copy region (SSC). The map was constructed using OrganellarGenomeDRAW. Figure S4. Amino acid frequencies in the chloroplast genomes of five Physalis species. Figure S5. Neighbor-joining (NJ) tree based on the complete chloroplast genome sequences of 36 species of Solanace [file 12870_2020_2429_MOESM2_ESM.docx]

**BMC Plant Biology**

**SUPPLEMENTARY INFORMATION FOR:**

**Complete chloroplast genomes of four *Physalis* species (Solanaceae): lights into genome structure, comparative analysis, and phylogenetic relationships**

Shangguo Feng^1, 2^, Kaixin Zheng^1, 2^, Kaili Jiao^1, 2^, Yuchen Cai^1, 2^, Chuanlan Chen ^1^, Yanyan Mao^1^, Lingyan Wang^1^, Xiaori Zhan^1, 2^, Qicai Ying^1, 2^ and Huizhong Wang^1, 2^ *

This file contains the supplementary information corresponding to figures, identified as such in the main manuscript. The order of the figures is kept as their first citation in the main text.


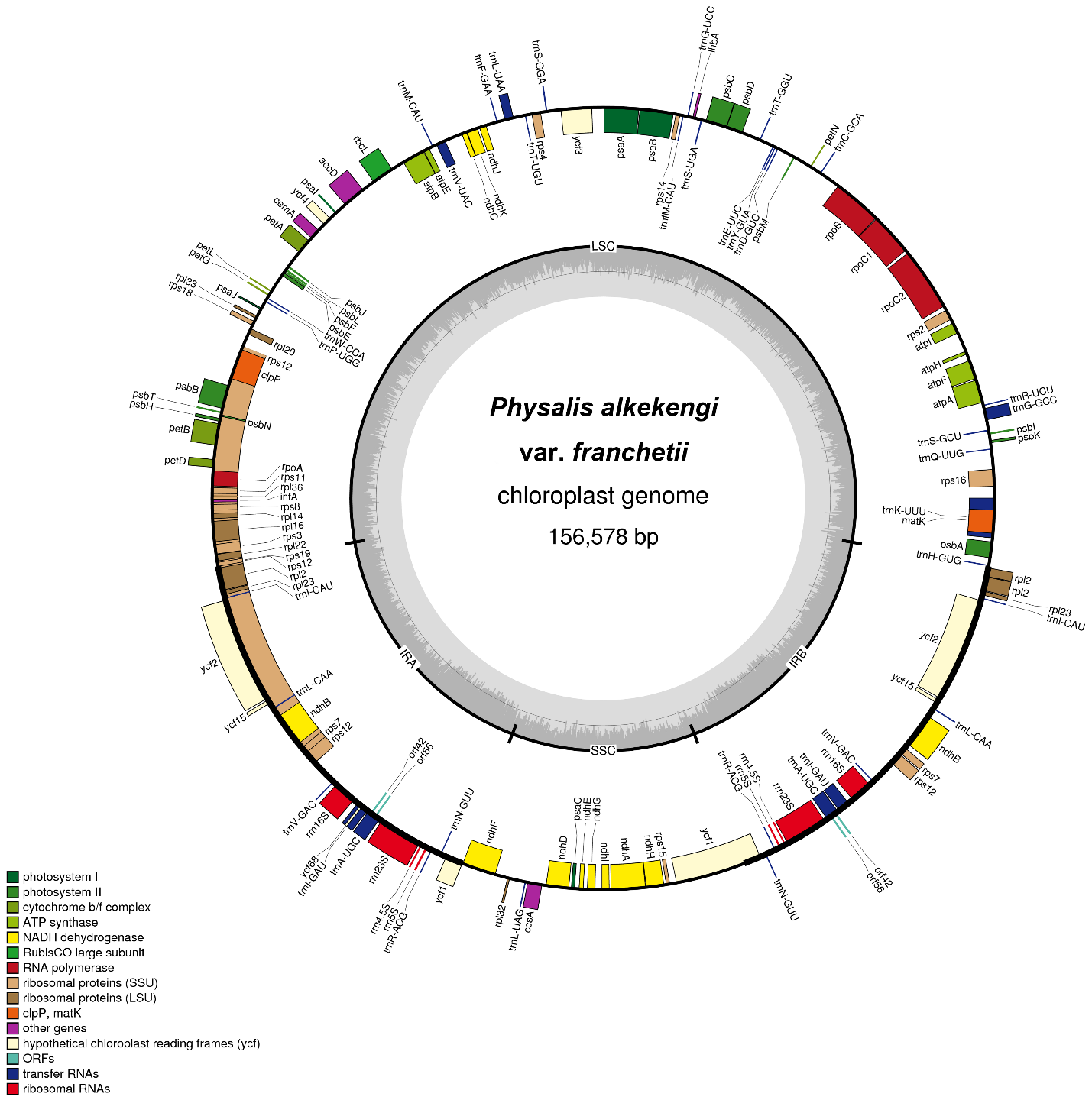


**Fig. S1** Gene map of the *P. alkekengi* var. *franchetii* chloroplast genome. Genes shown outside the outer circle are transcribed clockwise, and those inside are transcribed counterclockwise. Genes belonging to different functional groups are color coded. The darker gray in the inner circle indicates the GC content, and the lighter gray indicates the AT content. The inner circle also indicates that the chloroplast genome contains two copies of the inverted repeat (IRA and IRB), a large single-copy region (LSC) and a small single-copy region (SSC). The map was constructed using OrganellarGenomeDRAW.


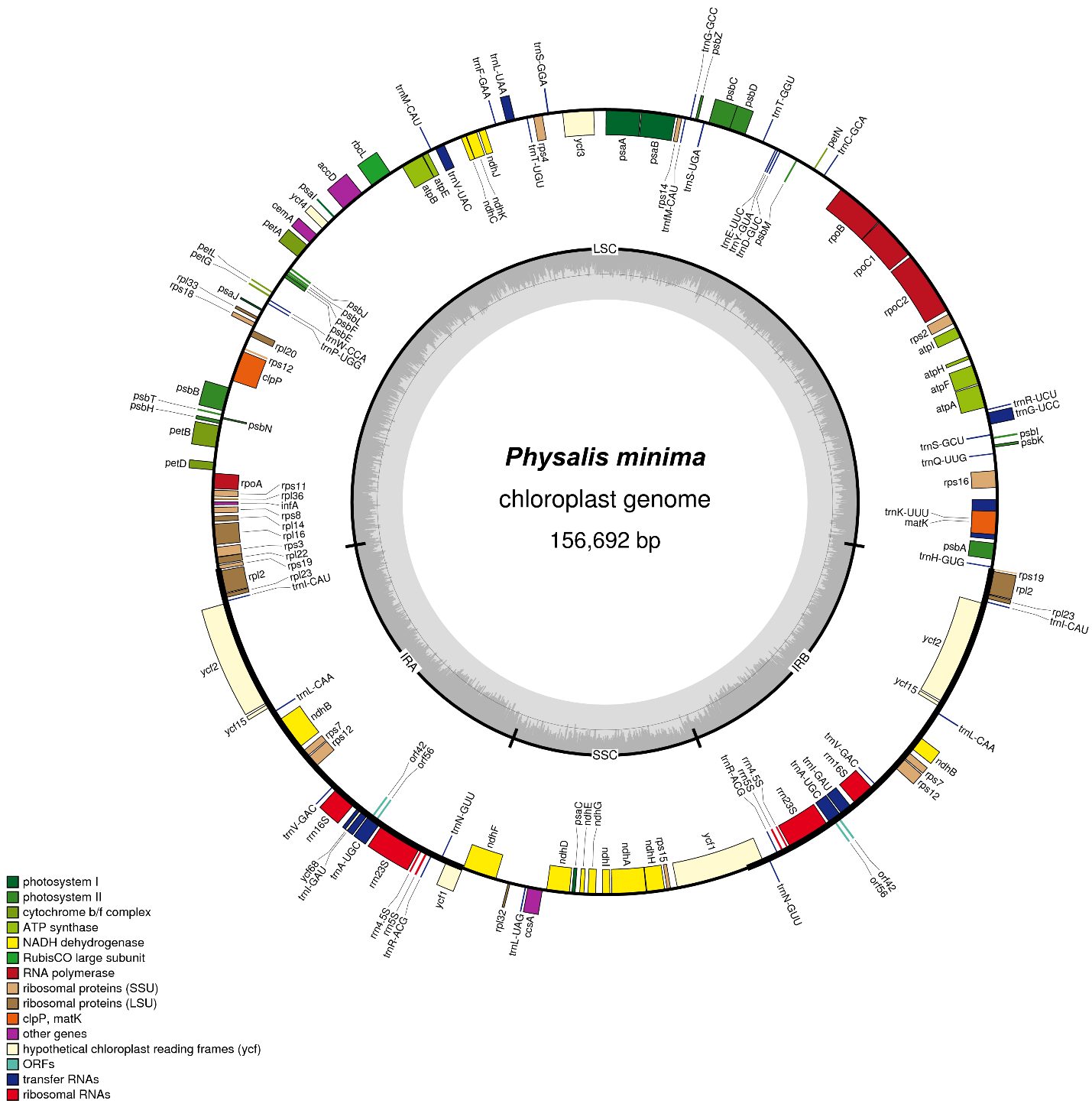


**Fig. S2** Gene map of the *P. minima* chloroplast genome. Genes shown outside the outer circle are transcribed clockwise, and those inside are transcribed counterclockwise. Genes belonging to different functional groups are color coded. The darker gray in the inner circle indicates the GC content, and the lighter gray indicates the AT content. The inner circle also indicates that the chloroplast genome contains two copies of the inverted repeat (IRA and IRB), a large single-copy region (LSC) and a small single-copy region (SSC). The map was constructed using OrganellarGenomeDRAW.


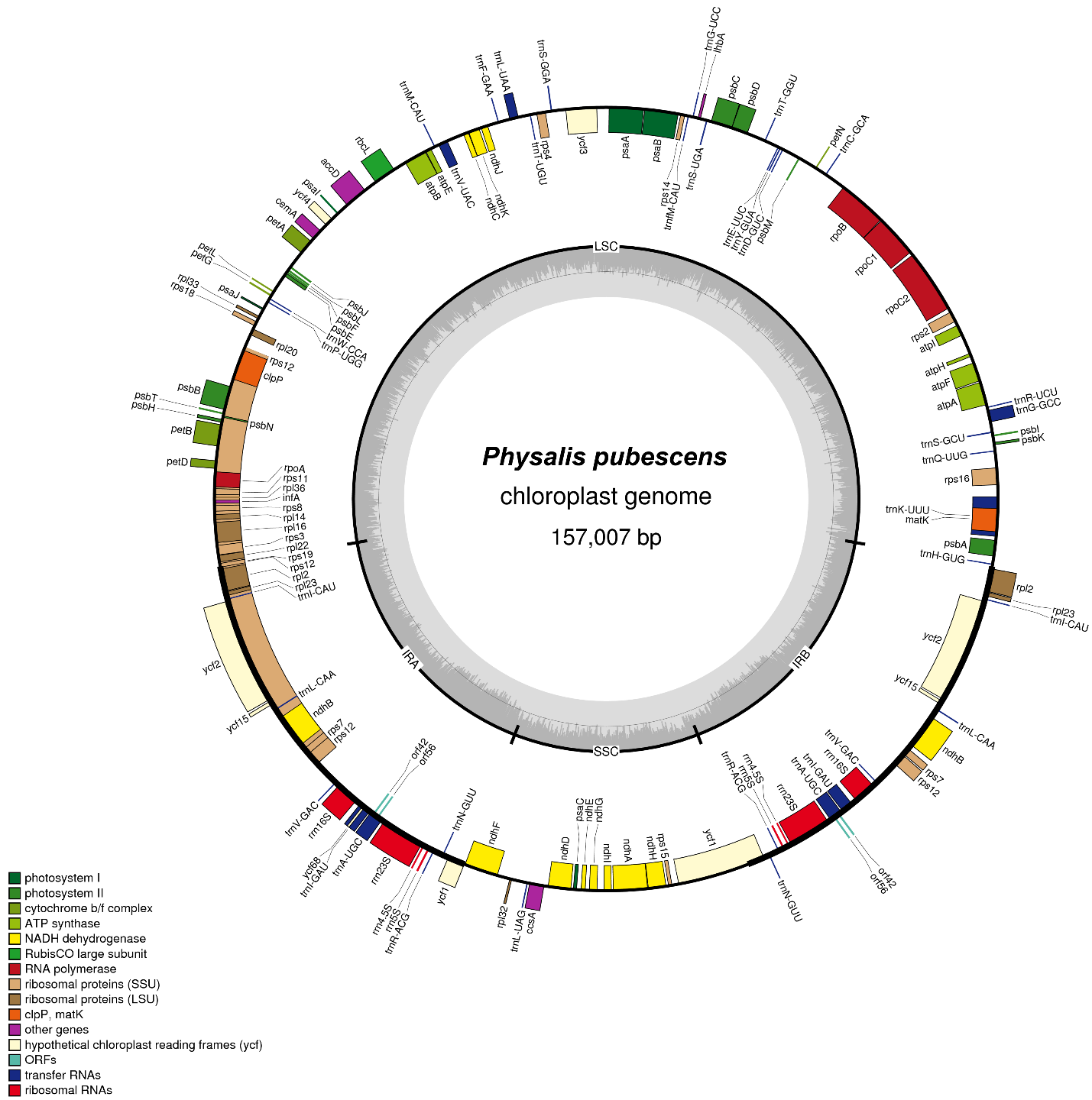


**Fig. S3** Gene map of the *P.* *pubescens* chloroplast genome. Genes shown outside the outer circle are transcribed clockwise, and those inside are transcribed counterclockwise. Genes belonging to different functional groups are color coded. The darker gray in the inner circle indicates the GC content, and the lighter gray indicates the AT content. The inner circle also indicates that the chloroplast genome contains two copies of the inverted repeat (IRA and IRB), a large single-copy region (LSC) and a small single-copy region (SSC). The map was constructed using OrganellarGenomeDRAW.


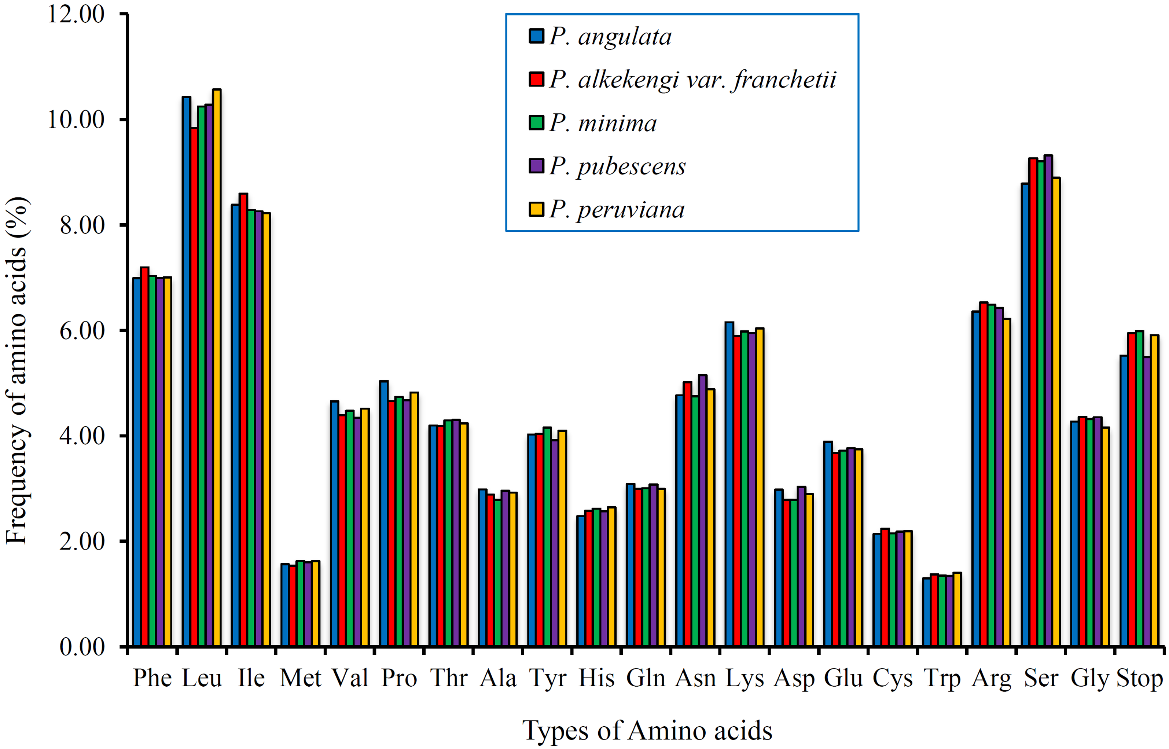


**Fig. S4** Amino acid frequencies in the chloroplast genomes of five *Physalis* species.


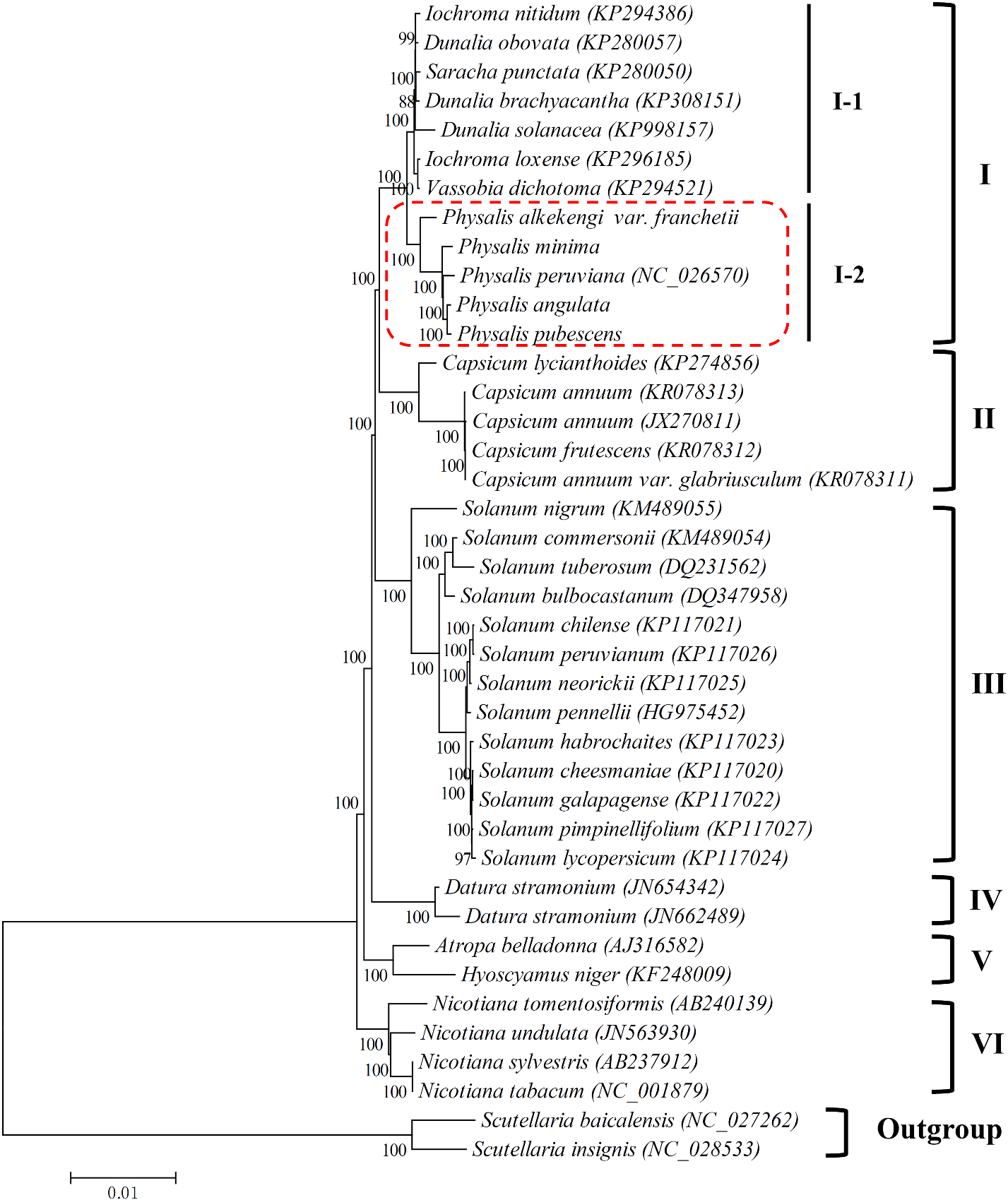


**Fig. S5** Neighbor-joining (NJ) tree based on the complete chloroplast genome sequences of 36 species of Solanaceae. Numbers above branches indicate bootstrap support, and circled by the red dotted lines are the five *Physalis* species.
